# Bednar tumour, an uncommon tumour of the skin: a report of two cases

**DOI:** 10.11604/pamj.2022.42.261.26498

**Published:** 2022-08-09

**Authors:** Tushar Kalonia, Aditi Dhanta, Ashok Singh, Arvind Kumar, Akanksha Malik, Neha Kumari

**Affiliations:** 1 Department of Pathology and Laboratory Medicine, School of Medical Sciences and Research, Sharda University, Greater Noida, Uttar Pradesh, India,; 2 Department of Dermatology, All India Institute of Medical Sciences, Rishikesh, Uttarakhand, India,; 3 Department of Pathology and Laboratory Medicine, All India Institute of Medical Sciences, Rishikesh, Uttarakhand, India,; 4 Department of Pathology, Sanjay Gandhi Postgraduate Institute of Medical Sciences (SGPI), Lucknow, Uttarpradesh, India

**Keywords:** Dermatofibromasarcoma protuberans, pigmented tumor, skin, case report

## Abstract

Pigmented dermatofibromasarcoma protuberans (DFSP) is a rare variant of DFSP which is an intermediate-grade tumour due to its tendency for local reccurence. Morphology of this variant impose a differential diagnosis that is must know for a pathologist. We describe two cases that presented with pigmented papulo-nodular form involving back and lower limb in middle aged to an elderly female. Histopathology coupled with immunohistochemistry yielded daignosis of Bedner tumour. A surgical histopathologist needs to have knowledge of all differential of pigmented neoplasm of skin just to ensure not to skip this rare entity.

## Introduction

Dermatofibrosarcoma protuberans (DFSP) is an intermediate-grade dermal connective tissue tumour with low malignant potential. It is a slowly growing, locally aggressive tumour which shows frequent recurrences. Dermatofibrosarcoma protuberans comprises 1% of skin malignancies and usually occurs in 3^rd^ to 4^th^ decade of life, however it can also occur in infancy [1]. Dermatofibrosarcoma protuberans have several histological variants based on morphologic features, however, there are no major prognostic differences between these variants. Pigmented DFSP (Bednar tumor) is one of the rare variant, first described in 1957 by Bednar under the name of storiform neurofibroma [2]. It accounts for less than 5% of dermatofibrosarcoma protuberans variants [3]. Other pigmented cutaneous spindle cell lesions like desmoplastic malignant melanoma, pigmented neurofibroma, melanotic schwannoma, neurocristic cutaneous hamartoma are considered in the differential diagnosis of pigmented DFSP. Immunohistochemistry and special techniques are essential for differentiating between these entities.

## Patient and observation

**Patients’ information:** patient-1 is a 34-year-old female, presented with pigmented nodular swelling in the left lower limb for the last 6 years. Patient-2 is a 70 year-old-female presented with the dermatology outpatient department (OPD). The lesions were in form of multiple tender nodules which were firm to soft in consistency. The patient also gave a history of excision of a similar nodule 1 year back. However, no histopathology report could be found. There is no family history of similar lesions in both patients.

**Figure 1:**
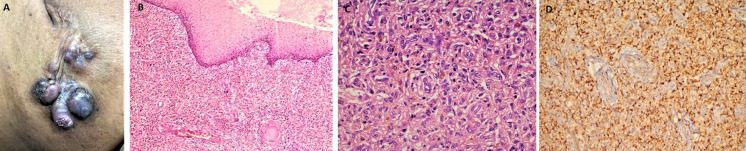
papulo-nodular pigmented, firm, fixed mass (gross); (A) dermis showing proliferation of monomorphic spindle shaped cell along with moderate infiltration of inflammatory cells and dilated congested blood vessels (H&E, 100X); (B) cells arranged in storiform pattern; individual cell showing oval to spindled vesicular nucleus with moderate to abundant eosinophilic cytoplasm; (C) scattered intra and extracellular brown pigment is also noted (H&E, 200X); (D) immunohistochemistry for CD34 is diffusely positive in neoplastic cells (IHC, 200X)

**Clinical findings:** patient-1 showed a papulo-nodular, pigmented, firm, tender swelling with restricted mobility (Figure 1 A). Patient 2 showed pigmented nodules on the left upper back for the past 3 months. Both patients showed within normal limits of blood pressure, respiratory rate and body temperature on clinical evaluation.

**Diagnostic assessment:** patient-1 underwent a punch biopsy measuring 0.6 x 0.5 x 0.5 cm for histopathology evaluation and patient-2 underwent a wide local excision of tumor measuring 5 x 4.2 x1.2 cm. The cut surface shows a greyish-white firm area. Microscopy of tumours of both patients was similar. Sections examined showed well-circumscribed tumours with cells arranged in a storiform pattern and thick collagen bundles amidst the tumour. Cells are spindle-shaped with moderate eosinophilic cytoplasm and vesicular nuclei. Many intermingled cells are showing cytoplasmic brownish orange coloured pigment granules obscuring the nuclear details also noted scattered extracellularly. Primarily benign spindle cell tumour diagnosis was kept with further characterization by immunohistochemistry (IHC) (Figure 1 B,C). A panel of IHC was performed and the tumor cells were positive for clusters of differentiation 34 (CD34) and negative for alpha smooth muscle actin (SMA), S-100, Human Melanoma Black (HMB)-45, Caldesmon, Melan A. Based on the histomorphology and immunohistochemistry, a diagnosis of pigmented dermatofibrosarcoma was rendered (Figure 1 D).

**Therapeutic interventions:** as a measure of therapeutic intervention a wide local excision/excisional biopsy with one c.m normal margins was done in both patients.

**Follow-up and outcome:** both patients were discharged postoperative day one with no complications. Patients were called for follow-up after one week.

**Patients’ perspective:** patients were satisfied with the treatment given and thankful to the treating clinician.

**Informed consent:** written consent was obtained from both patients.

## Discussion

Dermatofibrosarcoma protuberans is a dermal tumour which also infiltrates into the deeper subcutaneous tissue, fascia, muscle and sometimes even bone [4]. Initially, it presents as a slow growing brownish to a skin-coloured papule and progresses to a multinodular protruding mass. DFSP can occur at any location however trunk and shoulder regions are the most common sites [5]. Even though the histogenesis of pigmented DFSP is controversial, neuro-ectodermal differentiation or melanocytic colonization are the two proposed theories for histogenesis for the Bednar tumour. Some consider this tumour to be of neuroectodermal origin due to the presence of dendritic melanocytes and cells showing schwannian differentiation, while others believe its origin is due to local trauma like an insect bite, vaccination scars and previous burn sites [6]. It has also been reported in association with dermal melanocytosis (nevus of Ito), and based on the immunohistochemistry, the cell of origin is thought to be a neuro-mesenchymal cell. Bednar tumour can rarely undergo a malignant transformation that is fibrosarcoma with repeated recurrences and distant metastasis. The clinical differential diagnosis of DFSP in childhood include vascular malformations, keloids, scars, cystic hygromas, infantile myofibroma, pilomatrixoma, dermatofibroma, cellular blue nevus, fibrosarcoma, rhabdomyosarcoma, and malignant melanoma [7,8]. Various dermoscopic features of Bednar's tumour have been described. Bernard *et al*. reported six dermoscopic features: delicate pigment network (87%), vessels (80%), structure less light-brown areas (73%), shiny white streaks (67%), pink background coloration (67%) and structureless hypopigmented or depigmented areas (60%) [9]. In our case, dermoscopic observation showed homogeneous black-bluish pigmentation.

Microscopically, DFSP presents as a single well-circumscribed proliferation of spindle cells with mild nuclear pleomorphism and low mitotic count. The tumour cells are arranged in short irregular fascicles and storiform patterns similar to classical dermatofibrosarcoma but with scattered melanosome-containing dendritic cells. Certain unusual morphological features like prominent meningothelial- like whorls have also been reported in a case of Bednar tumour by Wang *et al*. [10].

Other rare variants of DFSP include myxoid DFSP contains abundant myxoid stroma. This type of tumour is uncommon, presents a diagnostic challenge and is important to recognize in order to prevent both under- and over-treatment. Giant cell fibroblastoma, referred to as juvenile DFSP affects children and adolescents, and is characterized by giant cells in the tumour [3]. It appears to be histologically similar to DFSP and in rare instances can be found within the same tumour in conjunction with DFSP, resulting in a hybrid lesion. Rarely, DFSP can have areas that look familiar to fibrosarcoma, a more aggressive type of soft tissue sarcoma. In these cases, the entity is called Fibrosarcomatous (FS) DFSP. These tumours are more likely to metastasize than other types of DFSP. Dermatofibrosarcoma can be distinguished from dermatofibroma by CD34, which is negative in the latter. Other entities in the differential diagnoses of DFSP are all negative for CD34 and show positivity for other immunohistochemical markers. Schwannomas and neurofibromas present very strong positivity for S-100; leiomyomas and leiomyosarcomas are positive for multiple smooth muscle markers (actin, desmin and vimentin), and the presence of positivity across the lesion for S-100, HBM-45 and Melan-A suggests a melanoma.

A high degree of clinical suspicion is required for diagnosing it at an early stage. Hence to overcome the usual delay in diagnosing this entity, Bednar tumour should be considered as a differential diagnosis in any long-standing, slowly growing pigmented skin tumour or nodular lesion, which does not spontaneously resolve. Bednar tumor may sometimes undergo fibrosarcomatous transformation with rare examples of pulmonary metastasis [11]. Bednar tumour presents a diagnostic challenge to the clinician because of its resemblance to other commonly occurring pigmented lesions. Through these two cases, we wish to describe the clinical and histopathological characteristics of DFSP and emphasize that Pigmented variants should be suspected in the presence of even a single papulo-nodular lesion, of slow and progressive growth. Diagnosis is through characteristic histopathology featuring spindle cells in a storiform pattern and positive immuno- histochemical study for CD34. Longer the delay in completely resecting the lesion, the higher the risk of recurrence.

## Conclusion

Bednar tumour needs to be recognized by the practising pathologist to differentiate from other cutaneous spindle cell neoplasm and close follow-up is always needed due to its frequent recurrence.
